# Influence of Streamer's Social Capital on Purchase Intention in Live Streaming E-Commerce

**DOI:** 10.3389/fpsyg.2021.748172

**Published:** 2022-01-24

**Authors:** Ping Xu, Bang-jun Cui, Bei Lyu

**Affiliations:** ^1^Department of Educational Psychology, School of Leisure Sports and Management, Guangzhou Sport University, Guangzhou, China; ^2^GuiZhou Vocational Technology College of Electronics & Information, Guizhou, China; ^3^School of Economics and Management, Huaibei Normal University, Huaibei, China; ^4^Chinese Graduate School, Panyapiwat Institute of Management, Nonthaburi, Thailand

**Keywords:** live streaming commerce, information asymmetry, social capital, trust, purchase intention

## Abstract

The virtual display of products in e-commerce brings new problems of information asymmetry, and the overload of digital information also increases the difficulty of consumers' purchasing decisions. The real-time interaction between the streamer and the consumer during live streaming e-commerce will promote consumers' understanding of the product, reduce information asymmetry, and increase consumers' purchase intention. However, why do people trust the untouchable and unfamiliar streamers from live streaming e-commerce to purchase online? To understand this phenomenon, based on the perspective of the information asymmetry theory and parasocial relationship theory, this research identified how social capital affected purchase intention in live streaming e-commerce. Through a questionnaire survey of live viewers, the purchase intention model constructed by empirical testing was used. The findings showed that the streamer's professionalism, the reciprocal expectation of live streaming, and the viewer's parasocial relationship could effectively increase the viewer's purchase intention. The occurrence of a streamer's negative public events could significantly reduce the viewer's purchase intention. The scale of live streaming and the streamer's commitment had no significant impact on the viewer's purchase intention. Trust played an intermediary role between the streamer's professionalism and parasocial relationship and the viewer's purchase intention.

## Introduction

The emergence of e-commerce has changed the way of exchanging traditional transaction information, fundamentally breaking the limitation of time and space, and providing traders with a powerful information search function. Buyers can obtain thousands or even more symbolic product information in a very short period (Bauboniene and Guleviciute, 2015). However, in e-commerce, there is the separation of information and physical objects, the separation of commodities and sales websites, and the separation of traders and physical spaces, which hinder the direct perception of trust between the two parties in the product quality and interpersonal relationship (Pavlou et al., 2007). In the network environment of e-commerce transactions, consumers' purchasing decisions not only depend on the final product quality but also depend on the network sales scene, environment, and content presentation form (Wongkitrungrueng et al., 2020). Based on the e-commerce commodity transaction process, it is necessary to solve the problem of the inability to transmit the actual product information caused by online sales, product quality information display fraud, and information overload, which prevents the bounded rational buyers from making deterministic judgments, which are different from the information asymmetry problem of traditional markets (Jones and Leonard, 2014). To increase consumers' purchase intention, e-commerce has continuously updated information release and transaction methods to reduce the information asymmetry of products, forming traditional transaction e-commerce, social e-commerce, content e-commerce, video content e-commerce, live streaming e-commerce, and other online sales patterns. E-commerce merchants use advertising, branding, celebrity endorsements, product trials, consumer reviews, 7 days of unreasonable returns, and other “signaling” methods to disclose their product information to gain consumers' purchase approval (Mavlanova et al., 2012; Filieri, 2015; Manes and Tchetchik, 2018). Live streaming e-commerce, as the advanced and the latest form of e-commerce, enables consumers to obtain virtual perceptions of commodities such as smell, taste, and touch through the alternative experience of the streamer. Live streaming e-commerce reduces consumers' transaction decision time through the “signaling” method of the streamer's narration of the product to a certain extent, solves the problem of the product quality perception and information overload, and effectively reduces information asymmetry between buyers and sellers (Wang et al., 2021).

The streamer is a connection point between the product and the consumer in live streaming. The streamer's image and word-of-mouth affect the live viewer's perception of the product. Live streaming viewers can easily derive trust in product quality based on the streamer's good reputation, which can effectively increase their purchase intention (Ang et al., 2018; Wongkitrungrueng et al., 2020). However, in actual purchases, it is found that “exaggerated words,” “exaggerated propaganda,” “false propaganda,” “use of advertising limit words,” and “difficult return and exchange” have become high-frequency words in consumer complaints (Hu and Chaudhry, 2020). Some studies found that in the process of live streaming, the emotional preference of the fan group for the streamer during live streaming will dilute the fact that the streamer is suspected of false propaganda. The actual product price, quality, etc., are not the main factors that affect consumers' purchases. Celebrity streamers are fundamental to attracting users (Clement Addo et al., 2021). Why consumers trust and buy the products sold by unfamiliar streamers in the virtual world of the network has become a hot issue of research. However, current research tends to focus on the information display and social interaction forms of live broadcast, as well as consumers' motivation to watch live streaming or purchase, and there is a lack of research on the reasons for the purchase intention of live streaming viewers (Han et al., 2018).

This research focuses on the perspective of information asymmetry combined with the social capital theory, reveals the role of the live streaming sales model and the streamer's social influence on reducing information asymmetry in the consumer purchase process and explains the impact of the streamer's social capital on the viewer's purchase intention to better understand the bond relationship of the streamer in live streaming. First, this research introduces the basic concepts of live streaming and discusses the mechanism of information asymmetry in live streaming commerce and the role of pseudosocial relations. Then, the social capital theory is used to establish a model to test how social capital promotes consumers' purchase intention, and empirical testing of this model is conducted through the survey data collected from consumer questionnaires who have participated in live streaming purchases. Finally, how the empirical results can contribute to the development of information asymmetry theory is discussed and the understanding of how the social capital of the streamer can promote consumers' purchase intention is improved.

## Literature Review

### Information Asymmetry in Live Streaming E-Commerce

Information asymmetry is the content of information economics. Compared with the traditional market, e-commerce has improved information efficiency and reduced information asymmetry in terms of the information that is suitable for digital transmission (Liang and Huang, 1998). However, for the product quality information in e-commerce, it is not suitable for digital delivery. Because quality information is closely related to the product itself, it can only be obtained by watching, touching, using, or testing. Due to the reproducibility of digital products, empirical digital products are also unable to convey their quality information (Chatterjee and Datta, 2008). Therefore, from the perspective of quality information transmission, the information efficiency of e-commerce relative to traditional markets has not improved, and information asymmetry still exists (Jones and Leonard, 2014). Based on the characteristics of physical distance and easy copying of digital information, e-commerce merchants have much more information about the products they sell than consumers, and the product is prone to cross-regional low-price competition and sell seconds at best quality prices among e-commerce merchants (Bakos et al., 2005). Consumers buy similar products of “high quality” and “bad quality” at lower prices. Buyers are only willing to pay according to the average price of the products on the market under the premise of being unable to distinguish the quality of the products. The final result is that “high-quality” products have been withdrawn from the market due to low profits, and “poor-quality” products flood the e-commerce market. Consumers' adverse selection leads to the phenomenon of “bad money drives out good” (Wei et al., 2011).

The e-commerce market is not only due to a new problem of information asymmetry brought about by the sales method, but also the digital information overload, which increases the time cost of consumers' purchase decisions under limited rationality (Cromer, 2011; Chen, 2019). The combination of e-commerce and live broadcasting is regarded as a way to solve these problems. The streamer recommends products in real time through on-site explanations, evaluations, etc., interacts with users in real time through the comment area, and uses the advantage of “face-to-face” communication to create a shopping experience close to offline, which greatly breaks the invisible, intangible, and unfeeling experience and information barriers with opaque prices (Zhang et al., 2020). The marketing model before the emergence of live streaming has standardized and one-way transmission of commodity information to consumers' purchase intention. The streamer can establish an emotional connection with consumers through personalized personality and infectious expressions, thus increase consumers' trust in the streamer and its recommended products (Wongkitrungrueng and Assarut, 2018). Trust also promotes the reliability of information transmission between the two parties in the market economy activities and reduces the moral hazard and adverse selection caused by information asymmetry in market transactions (Liu et al., 2019). Live streaming e-commerce can be understood as the online sales behavior of live streaming using their influence and a certain viewer base. The net connection between “people” and “consumers” can effectively promote consumers' willingness to watch. The streamer has an important dominant position in live streaming e-commerce (Hu et al., 2017). Live streaming solves the problem of information overload to a certain extent. As an intermediary between sellers and consumers, streamers understand the product's efficacy in advance and make recommendations after the trial screening, which will reduce the asymmetry of information between buyers and sellers and reduce the time cost for consumers to purchase (Xu et al., 2020). As an upgraded model of traditional e-commerce, live streaming e-commerce can overcome the trust problem caused by information asymmetry in the traditional model and facilitate transactions. When understanding the influence of the streamer in live streaming e-commerce, we need to have an understanding of the role of the social capital theory in sales promotion.

### Live Streaming E-Commerce and Social Capital

Social capital is usually defined as “resources embedded in the social structure that can be accessed or mobilized in purposeful actions” (Lin, 2001). The concept of social capital has been provided as an explanation for various pro-social behaviors, including the concept of collective action, community participation, and differential social achievement, which is based on personal capital (such as human or financial capital) that cannot be explained (Coleman, 1990). Social capital that is embedded in the social realm is a key difference from other forms of capital. Social capital is widely considered to be dualistic: at the group level, it reflects the nature of emotions and the quality of relationships, while at the individual level, it promotes the actions of actors and reflects their access to social network resources (Putnam, 1995). From an individual's perspective, social capital is the position and identity that an individual occupies in the social network of the social structure. These positions and identities play an important role in the actual goals of an individual.

Live streaming e-commerce is in radiant communication with the streamer as a central node. The streamer plays an important role in live streaming e-commerce, highlighting, and responding to the importance of people as a central node of communication (Hu and Chaudhry, 2020). The streamer and the viewer may be “never knew each other at all.” The streamer's personality charm, social influence, and interactive communication during live streaming have become the core link to maintain consumer relations. The social capital of the streamer's position and identity in the entire social network plays an important role in consumers' trust. Nahapiet and Ghoshal (1998) demonstrated that social capital with shared functions, high interdependence, frequent interaction, and closed structure is more likely to develop in the collective. Therefore, the user interacts with the streamer by launching or watching live streaming, expressing emotions similar to others, and showing similar experiences, to achieve emotional resonance and form a sense of group identity (Lin et al., 2021). From the perspective of theoretical logic, the social capital level of the streamer can easily establish trust in a relationship with the viewer to facilitate the purchase, which has a significant effect. The current research does not involve the correlation between significant levels of social capital and consumers' purchase intentions in live streaming. Therefore, the key question that this research attempts to solve is whether streamers in live streaming can improve their social influence and the emotional rendering ability in the live broadcast process can promote consumers' purchase intentions. How to improve the influence of the streamer in a virtual social network, to build a close relationship with consumers, should focus on the pseudosocial relationship factor.

### Live Streaming E-Commerce and Parasocial Relationship

The concept of parasocial relationships was first proposed by Horton and Wohl (1956). At that time, they observed that people began to interact with TV characters in mass media such as TV, and most people felt that there was a real social relationship. This “phantom of one-way social relationship” was called a parasocial relationship. In psychology, the most common scenario of parasocial relationships is used to describe fans' admiration for celebrities, which can also be understood as the “star effect.” According to this logical extension, scholars mostly pay attention to the “virtual” existence in one-way relationships and call the phenomenon of virtual intimacy a parasocial relationship (Rubin and Step, 2000). Celebrities are widely promoted because of their achievements in their respective fields. The parasocial relationship groups of celebrities often imitate the style and taste of celebrities and are more willing to trust the opinions of celebrities. At the same time, users of parasocial relationships will actively use media channels to obtain information about intimate relationships and engage in interactive activities as much as possible to pursue satisfaction (Palmgreen et al., 1980). The development of internet media incorporates the concept of parasocial relations, and its social and broadcast media applications have produced some new pseudosocial relations vocabulary, such as idols, internet celebrities, clout, and popularity. The parasocial relationship is directed to followers with virtual intimacy. They admire individuals who have a certain degree of influence in a virtual platform. From the perspective of social structure, parasocial relationships belong to the social capital of such “star” groups.

Streamers are the central figures of live streaming commerce programs, their flow rate and popularity can quickly gather consumers and achieve higher sales performance (Clement Addo et al., 2021). Therefore, celebrities, online celebrities, key opinion leaders, MCs, celebrity entrepreneurs, government officials, etc., who have a large number of pseudosocial relationship users, have used live broadcast platforms to recommend products to promote sales (Li et al., 2020). Consumers with parasocial relationships not only trust the streamer due to their strong emotional connection but also evaluate the streamer's credibility through the media and netizens outside live streaming (Bapna et al., 2017). If there are negative events such as “False propaganda” and “Poor quality” related to the product during live streaming of the streamer, or an even negative social public opinion that has nothing to do with the delivery activity, it will reduce consumers' trust and perception of the streamer and directly affect consumer's willingness to watch live streaming, consumers' trust, and repurchase rate. People will predict future behavior based on previous behavior. These beliefs form the basis of reputation, which is also regarded as the result of long-term consistent evaluation based on behavior. In normal interpersonal relationships, the parasocial relationship of the streamer is the performance of consumers based on a comprehensive review of their long-term accumulation of reputation information. How consumers who watch live streaming choose between the streamer's occasional negative events and the long-term parasocial relationship, and whether the streamer's social capital can form the mitigation of a few negative events have become other topics of this research. Based on the theoretical model proposed by Nahapiet and Ghoshal (1998), this research proposes a series of hypotheses to test how three forms of social capital (cognition, structure, and relationship), parasocial relationships, and negative events affect viewers' buying intentions in live streaming.

## Hypothesis

**Figure 1:**
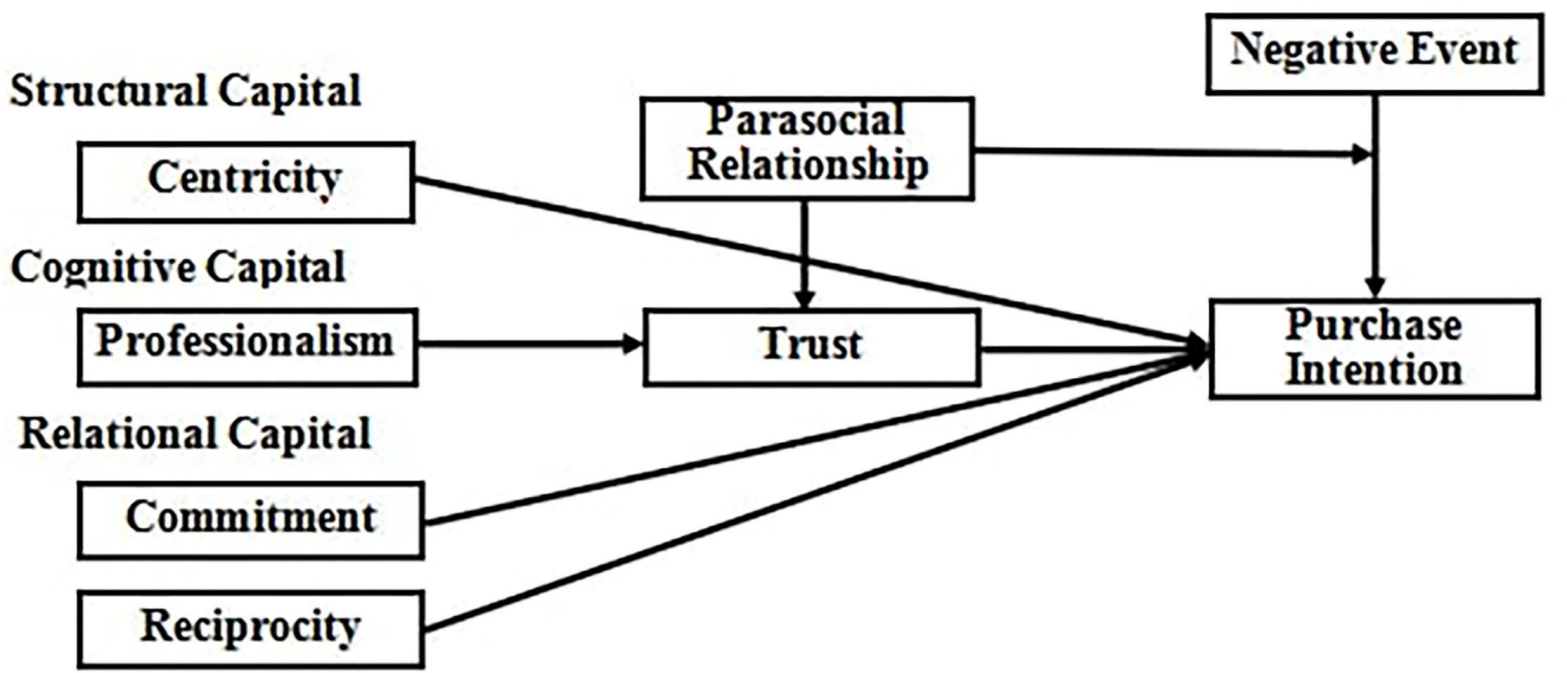
Research framework.

Social capital proposed by Nahapiet and Ghoshal (1998) includes structural links or connections between individuals (structural capital), personal cognitive ability to understand and apply knowledge (cognitive capital), strong relationships between them, and positive characteristics (relational capital).These forms of social capital constitute an aspect of the social structure and promote the exchange of information between individuals within the structure. Although the model of Nahapiet and Ghoshal (1998) focuses on social capital factors at the group level, research has found that social capital can also explain the sales promotion relationship at the individual level in live streaming e-commerce. Live streaming e-commerce brings goods through the technology platform to integrate the individuals and collectives participating in the interaction and sales relationship in live streaming as a whole. These personal relationships are the main sources of the social capital of the streamer, which affects how consumers trust the streamer's behavior to have a purchase relationship and promote the sales performance of live streaming e-commerce. Structural capital evaluates the network density and centralization of the entire organization and applies it to the personal level of the streamer. The degrees of centralization and neutralization of the streamer in live streaming e-commerce are used to measure the purchase intention formation due to the influence of the streamer. At the same time, it evaluates how the degree of the streamer's cognitive capital and the perception of relationship capital affect purchase intention of live streaming viewers. The research hypothesis model is shown in Figure 1. Each structure and its relationship with purchase intention are described in Section Structural Capital Hypothesis.

### Structural Capital Hypothesis

Structural capital is a structural link created by personal connections in a network through social interactions and is an important predictor of collective action (Burt, 1992). When the social network is dense, the more individuals are in constant contact, the more likely it is to conduct collective actions and obtain more information exchange (Marwell and Oliver, 1988). In a group, individuals embedded in the group at the center have a higher proportion of direct contact with other members. These individuals are more likely than others to understand and abide by group norms and expectations and to gain the recognition and trust of others (Rogers and Kincaid, 1981). The centrality measurement evaluates how many unique individuals (consumers) are connected to a focal individual (streamer), and the structural capital is evaluated by determining the degree of centrality (scale) of an individual streamer to a network. Therefore, the centrality of the streamer's structural position in live streaming is reflected in the number of viewers that the streamer has attracted. Judging from the fact that celebrities, government officials, and other public figures with a high social influence have resorted to living streaming platforms to recommend products for sale, the size of the streamer represents its popularity. In the process of live e-commerce, public figures rely on their strong influence and popularity to help live e-commerce complete the trust “endorsement,” dispel some consumers' doubts about live e-commerce and greatly improve the credibility of live streaming e-commerce. An all-around display of products with live streaming and the communication and interaction between consumers and streamers greatly increase the disclosure of product information. The larger the live streaming, the smaller the asymmetric information of the product, the higher the trust of consumers in the product and the streamer, and the stronger their willingness to buy. At the same time, “celebrities” with a high social influence carry out product recommendations and sales. The parasocial relationship they bring to consumers maximizes the social value of products, which has a positive impact on product sales and stimulates consumers' purchase intention (Park and Yang, 2010). The higher the streamer's parasocial relationship, the larger the broadcast scale, the more comprehensive the supervision of the platform and all sectors of the society, the more positive the media or the netizens outside the live broadcast room, the more consumers' trust in the streamer, and the more trustworthy it is. It acts as a bridge in enhancing the purchase intention of the product. Hypotheses 1–3 are listed as follows.

H1: Streamers with a high level of centrality in live streaming increase the willingness of live streaming viewers to purchase intention.
H2: Streamers with a higher parasocial relationship in live streaming increase the purchase intention of live streaming viewers.
H3: Trust has an intermediary effect in the influence of the parasocial relationship of live streaming viewers on purchase intention.

### Cognitive Capital Hypothesis

Cognitive capital refers to the resources that enable information to have a shared interpretation in the collective (Taegoo et al., 2013). Engaging in a meaningful knowledge exchange requires at least some degrees of shared understanding between the parties, such as shared language and vocabulary. Individuals' cognitive capital develops along with the time they interact with others to share the same or practice of learning skills, knowledge, and professional discourse. That is, cognitive capital includes both personal professional knowledge and experience in applying professional knowledge. During the live streaming e-commerce process, the streamer plays a full-scale role in the consumer's purchase experience, such as shopping guides, model trials, and customer service teaching. The full range of online merchandise marketing is mapped to the consumption scenarios of offline physical stores, and consumers' sense of experience and satisfaction has been greatly improved. The streamer's comprehensive and detailed introduction to the product reflects “professionalism.” The streamer explains product function and its usage information in a short period, which not only reduces the time and cost for consumers to understand the product on their own but also ensures the “credibility” of the product quality, and increases consumers' favorability and trust in the streamer and its recommended products. The dissemination situation and activities of live streaming commerce stimulate the viewers' emotional response and purchase behavior, and the emotional transmission of the streamer has also become a basic ability and skill (Douglas Pugh, 2001). Some researchers have elaborated the influence of the streamer's emotions on the viewer's emotions in interactive and dynamic business settings. For example, the use of symbolic images, virtual personas, and carefully constructed relationships can enhance viewers' consumption behavior (Lin et al., 2021). Therefore, the higher the streamers' professional knowledge or emotional skills, the more they can gain the trust of live streaming viewers, thereby increasing their willingness to buy. Hypotheses 4 and 5 are listed as follows.

H4: The streamer with a higher level of professionalism in live streaming e-commerce will significantly increase consumers' purchase intention.
H5: Trust has an intermediary effect in the influence of the professionalism of the streamer on the purchase intention of consumers.

### Relational Capital Hypothesis

Relationship capital refers to the collective emotional relationship (Wasko and Faraj, 2005). When members have a strong sense of identity with the group, they will trust other people in the group, believe that they are obliged to participate in the group and recognize and abide by its cooperation norms. Relationship capital is formed finally (Lewicki and Bunker, 1996). Coleman (1990) proposed that the relational aspect of social capital is the structure that promotes individual action. Relationship capital is an important asset, which is conducive to an emotional connection between community groups and their members. This research studies the content related to live streaming, including two dimensions in relational capital: commitment and reciprocity (Arli et al., 2018).

The commitment represents a responsibility or an obligation to participate in future actions and is generated by a frequent interaction (Hess and Story, 2005). Although commitment is often described as a direct expectation that arises in a specific personal relationship, it can also be accumulated into a collective organization. The goods-carrying streamer plays the role of artificially screening products, while live streaming viewers can easily derive trust in product quality based on the good reputation of the streamer. The related performance of the streamer during live streaming directly affects consumers' pre-purchase expectations. If there is a big difference between the experience after purchase, the streamer's reputation will be reduced. On the contrary, if the trust of the streamer is damaged, the first thing that will be affected is the lowered perception of the quality of the products that the streamer carries. Based on this, the streamer pays more attention to the sense of responsibility and moral obligation and often adopts the way of promise during the live streaming process to strengthen the live streaming viewer's understanding of the product and trust in themselves. If the live streaming streamer shows a strong sense of commitment during the live streaming process, it is more likely to increase purchase intention of the live streaming viewers. However, in reality, the live broadcast e-commerce industry has an uneven quality of streamers. Some live streaming e-commerce companies will conduct false propaganda and use consumers' trust to mislead consumers to make purchases. After such negative incidents broke out, consumers believed that they had been deceived and produced a strong resistance to the streamer, which seriously affected consumers' willingness to buy. The emotional identification of the fan group has a parasocial relationship with the streamer in the live streaming, which causes the fan group to adopt the “problem transfer” method to seek explanations for the idol streamer (Liebers and Schramm, 2019). Hypotheses 6–8 are listed as follows.

H6: The streamer shows the obligation of commitment during the live streaming process will further enhance the purchase intention of the live streaming viewers.
H7: Negative incidents of the streamer in live streaming commerce will reduce the purchase intention of live streaming viewers.
H8: The parasocial relationship between viewers and the streamer regulates the relationship between the negative events of the streamer and the viewer's purchase intention.

A basic norm of reciprocity is a sense of mutual indebtedness so that individuals usually reciprocate the benefits they receive from others, ensuring ongoing supportive exchanges (Shumaker and Brownell, 1984). Even though exchanges in live streaming e-commerce occur through weak ties between the streamer and viewer, there is evidence of reciprocal supportiveness (Wellman and Gulia, 1999). As the development form of e-commerce, live streaming e-commerce has captured the user's mentality of pursuing low-price and high-quality goods from the beginning of its rise (Wu et al., 2008). From the perspective of a communication strategy, real-time interaction is a significant feature of live streaming. In addition to the regular product explanation, Q&A, and lottery interactions, the streamer will also participate in the SecKill, grab red envelopes, and give gifts to the viewer to obtain support from live viewers (Rodriguez et al., 2014). There are always large numbers of statements such as “If you don't buy the products sold in live streaming e-commerce, you will lose the money.” The streamer recommended product “inexpensive and good in quality” has also strongly attracted live viewers to purchase. Therefore, when there is a strong standard of reciprocity in live streaming commerce, live streaming viewers believe that the products they buy are the most favorable, and thus have a higher purchase intention. This leads to the final hypothesis below.

H9: Under the guidance of the reciprocity rule, the live streaming e-commerce activity will increase the consumer's purchase intention.

## Research Design

### Sample Selection

The sample of research came from consumers who have watched live streaming. The data were collected through the internet and social networks in the form of questionnaire surveys. Since it was impossible to accurately confirm whether the respondent has watched the live streaming show, one question item “Have you ever watched the live streaming show” was placed next to the basic information of the respondent. If the respondent chose not to watch the live streaming, the following items will be hidden and displayed, and the respondent can choose to submit the questionnaire. Finally, the data of respondents who have watched live streaming were used for analysis. To solve the potential limitations of occupational categories brought about by the questionnaires issued by social groups, the questionnaires were distributed for different occupational groups in a purpose-based sampling method, focusing on the company teams and student groups that can be reached. Participants in the survey were all voluntary, and the questionnaire was spread and filled out in social applications such as WeChat and QQ. The questionnaire survey lasted for 1 week. After the time was over, the questionnaire data were collected and checked for validity.

A total of 551 questionnaires were collected, and no missing data or abnormal observations were found, and all were confirmed to be valid. The number of people who watched live streaming was 230, accounting for 41.74% of the total sample; the number of people who did not watch live streaming was 321, accounting for 58.26% of the total. Among the survey participants, there are 219 men and 332 women. The proportion of viewers was 46.69% for women, which is higher than 34.25% for men. The occupational categories of the respondents were more comprehensive, and the number of respondents was mainly concentrated in professional and technical positions (102 people), business units (66 people), government departments (52 people), and student groups (252 people). Although the number of service positions was the smallest, the proportion of watching live streaming was the highest, reaching 66.67%. A slight deviation from our understanding was that 37.89% of the student population watched live streaming, which was the lowest level among all categories. Respondents' education levels were mainly concentrated in colleges (226), undergraduates (173), and masters (101), while groups with education levels below high school have the highest percentage of watching live streaming, reaching 86.96%, followed by doctoral groups, as 50%.

The age group was relatively concentrated among the respondents. In the 21–30 age group, there were 310 people and 40.97% of them watched live broadcasts. Although the 41–50-year-old group (64.71%) has the highest percentage of watching live broadcasts, the total number was relatively small, only 17 people. No work experience (198 people), 0–3 years of work experience (164 people), and more than 8 years of work experience (104 people) accounted for 33.04, 30.87, and 17.39%, respectively, of the total number of viewers. However, judging from the viewing ratio of each category, it was smaller than the other two groups. Respondents' salary survey data, shows that the proportions of viewers in each category were 36.31, 42.31, 51.06, 45.07, and 34.04%, respectively. The proportion of people watching live streaming first rose and then started to decline with an increase in income. The overall sample composition is shown in Table 1. In a subsequent analysis of this study, the sample data (*n* = 230) of watching live streaming e-commerce programs were chosen.

**Table 1:** Demographic information of respondents (*N* = 551).

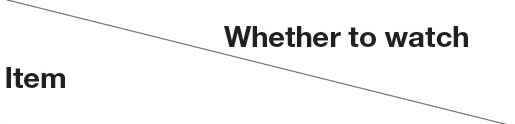		**No**	**Yes**	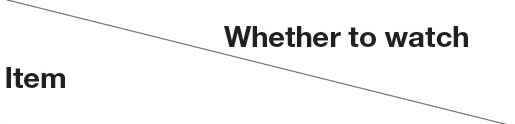		**No**	**Yes**	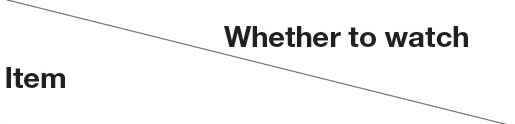		**No**	**Yes**
**Gender**	Male	144	75	**Education level**	High school and below	3	20	**Work experience**	Less than 3 years	122	76
	Female	177	155		College Degree	130	96		0–3 year	93	71
**Occupation category**	Technical position	54	48		Bachelor Degree	108	65		3–5 year	16	22
	Service position	4	8		Master Degree	66	35		5–8 year	26	21
	Worker	6	4		Doctoral Degree	14	14		More than 8 years	64	40
	Corporate employee	39	27	**Age**	20 year-old below	60	44	**Salary**	Less than ¥1,000	100	57
	Government staff	31	21		21–30 year-old	183	127		¥1,000–3,000	105	77
	Freelance	21	17		31–40 year-old	69	44		¥3,000–5,000	46	48
	Entrepreneur	7	8		41–50 year-old	6	11		¥5,000–10,000	39	32
	Student	159	97		51 year-old above	3	2		More than ¥10,000	31	11

### Variable Measurement

The measurement of the variables in this study was mainly based on the published measurement scales and items, and combined with the characteristics of live streaming e-commerce, the measurement items of each variable was designed. The measurement of centricity index adopted the Social Interaction Connection Scale of Chiu et al. (1993). According to the situation prevailing in live streaming e-commerce, the more viewers watching the streamer, the more consumers are connected to the streamer, and the higher the neutralization of the streamer, a single-item scale is designed to measure live viewers. The professionalism index adopted the scale of common language and a common vision of Kate (2010) and Cohen and Prusak (2001) combined the application context of live streaming e-commerce and designed a professionalism measurement scale including two items that describe the language expression and empathy ability of the streamer when explaining products. The scale for measuring commitment was adapted from the existing research results of Mowday et al. (1997) including three items. The reciprocity measurement was adapted from the scale used by Wasko and Faraj (2005) including two items.

The measurement of trust was adapted from the trust scale of McAllister (1995). The eight-item scale had two parts: cognitive trust and emotional trust. In the process of structural equation confirmatory factor analysis, according to the modification indices (MI) value of the initial model, to revise and adjust. Finally, the four items related to this research were retained. The measurement of parasocial relationships adopted the six-item questionnaire of Kim et al. (2015). In the confirmatory factor analysis, two items were deleted to modify the model. Negative incidents were designed with two-value variables for questionnaire design, mainly to obtain respondents' negative perceptions of whether there was false propaganda on the streamer. In this study, purchase intention was a dependent variable. The two-item scale from the purchase intention scale by Jiang et al. (2010) was adapted to include enquiring about the purchase intention and purchase decision of the viewer during live streaming, to measure the viewer's possibility of purchase during live streaming commerce.

According to the potentially influencing relationship of purchase intention, this research used gender, age, monthly disposable income, and platform heterogeneity as control variables. The current live streaming commerce platform was divided into five levels, from 1 to 5 representing the degree from weak to strong based on factors such as platform popularity, operating time, platform scale, platform background, and platform positioning. Except for gender and negative events, which use the binary measurement of accidents, other variables are measured using Likert's five-level scoring method, using 1–5 points as a scale to measure the degree of the consent of live broadcast viewers to the problem or the increase in age and income. Among them, 1 point represents a very low level and 5 points represent a very high level.

## Data Analysis

### Model Measurement

As the questionnaire survey belongs to self-reported data, it is necessary to conduct a series of confirmatory factor analyses of latent variables to determine their validity. This study applied the Maximum Likelihood Estimation method to analyze the multifactor correlation between items by using the variance adjustment estimator in Mplus. Previous studies have used this method to perform a CFA analysis on Likert-type data (Luksyte et al., 2020). Based on this method and the suggestion of a suitable fitting index, the fitting data of the six-factor model were: *x*^2^ = 517.75; *df* = 215; *p* < 0.01; Root Mean Square Error of Approximation (RMSEA) = 0.078, 90%; CI = [0.07, 0.09]; Comparative Fit Index (CFI) = 0.87; and Tucker Lewis Index (TLI) = 0.85. Since the value of *x*^2^/*df* < 3, RMSEA < 0.05 indicates that the model fits well, 0.08–0.10 fits well, >0.10 fits poorly, while CFI and TLI >0.95 and >0.90 indicate a good fitness level and an acceptable fit, respectively.

It could be seen from the model fitting results that the model fitting was not ideal and the model needed to be revised. As the data are linked, changing any one parameter will cause the entire variance–covariance matrix to change. Therefore, in the process of model modification, only one parameter was modified at a time, usually starting with the largest modification index. According to the maximum value of MI of the initial model, it was adjusted sequentially, and some items of parasocial relationship and trust were deleted. Finally, the model fitted values were *x*^2^ = 155.64; *df* = 89; *p* < 0.01; RMSEA = 0.05, CI = [0.04, 0.07]; CFI = 0.94; and TLI = 0.92. The model showed a good fit.

Table 2 shows the descriptive statistics and correlation coefficients of the variables. As the reliability coefficient of the total scale is preferably more than 0.8, with the acceptable limit being 0.7–0.8; the reliability coefficient of the subscale is preferably more than 0.7, with the acceptable limit being 0.6–0.7. The Cronbach's alpha coefficient of each scale in this study was >0.6, and the coefficient of the total scale was 0.806, indicating that the scale of this study has good reliability.

**Table 2:** Descriptive statistics and correlation coefficients of the variables.

**Variables**	**1**	**2**	**3**	**4**	**5**	**6**	**7**	**8**	**9**	**10**	**11**	**12**
1. Gender	—											
2. Age	0.222^**^	—										
3. Income	0.189^**^	0.441^**^	—									
4. Platform	−0.349^**^	−0.109	−0.030	—								
5. Centricity	−0.117	−0.154^*^	0.081	0.244^**^	—							
6. Professionism	−0.205^**^	0.063	0.047	0.154	0.245^**^	**0.727**						
7. Commitment	−0.047	−0.005	0.040	0.073	0.124	0.093	**0.623**					
8. Reciprocity	−0.053	−0.039	0.128	0.232^**^	0.118	0.066	0.545^**^	**0.637**				
9. Trust	−0.112	0.013	−0.051	−0.010	0.149^*^	0.486^**^	−0.093	0.016	**0.793**			
10. Parasocial	0.003	0.084	−0.062	−0.003	0.075	0.303^**^	−0.050	−0.067	0.560^**^	**0.805**		
11. Non-event	0.096	0.038	0.041	−0.010	−0.033	−0.212^**^	0.174^**^	0.215^**^	−0.194^**^	−0.137^*^	—	
12. Intention	−0.099	0.133^*^	−0.058	0.138^*^	0.141^*^	0.366^**^	0.015	0.110	0.556^**^	0.552^**^	−0.187^**^	**0.731**
*M*	0.326	2.130	2.448	4.108	4.583	3.261	2.938	2.980	2.991	2.842	0.517	3.109
*SD*	0.470	0.804	1.202	0.857	2.198	0.660	0.858	0.966	0.650	0.651	0.500	0.767

*N = 230, α-coefficient is bolded along the diagonal;*

** *p < 0.01,*

* *p < 0.05*.

### Hypothesis and Model Testing

#### Main Effect and Moderating Effect Test

Table 3 shows the regression analysis results of the estimated main effects and moderating effects. Model 1 was the parasocial relationship without a moderating variable; Model 2 was the moderating variable added; and Model 3 was an interaction item with the addition of negative events and parasocial relationship. Since it was assumed that the parasocial relationship had a significant impact on the purchase intention of live streaming viewers, the main effect test was mainly based on the regression results of Model 2. The adjusted *R*^2^-value of the model is 0.384, indicating that the model has a certain explanatory power. H1 proposed a positive connection between the streamer's centrality and purchase intention of live streaming viewers. The hypothesis testing results of H1 were not significant (β = 0.032, SE = 0.26, *t* = 1.42). H2 proposed a positive connection between the streamer's parasocial relationship and the purchase intention of live streaming viewers. The hypothesis testing results of H2 showed that the streamer's parasocial relationship significantly affected the purchase intention of viewers (β = 0.556, SE = 0.065, *p* < 0.01). H4 proposed that the professional level of the streamer affected the purchase intention of live streaming viewers. The regression results of H4 showed that the professionalism of the streamer positively affected the purchase intention of live viewers (β = 0.343, *SE* = 0.076, *p* < 0.01).

**Table 3:** Regression analysis of estimated main effects and moderating effects.

**Independent variable**	**Dependent variable: purchase intention (Inten)**		
	**Model 1**	**Model 2**	**Model 3**
Gender	−0.010	−0.059	−0.054
	(−0.09)	(0.095)	(0.095)
Age	0.221^**^	0.168^**^	0.161^**^
	(0.066)	(0.058)	(0.059)
Income	−0.123^**^	−0.082^*^	−0.082^*^
	(0.044)	(0.039)	(0.039)
Platform	0.045	0.054	0.051
	(0.75)	(0.052)	(0.052)
Centricity	0.032	0.024	0.023
	(0.026)	(0.020)	(0.020)
Professionism	0.343^**^	0.179^**^	0.185^**^
	(0.076)	(0.068)	(0.069)
Commitment	−0.085	−0.063	−0.058
	(0.065)	(0.056)	(0.056)
Reciprocity	0.146^*^	0.154^**^	0.152^**^
	(0.059)	(0.052)	(0.052)
Non-event	−0.221^*^	−0.175^*^	0.191
	(0.097)	(0.084)	(0.372)
Parasocial		0.556^**^	0.626^**^
		(0.065)	(0.095)
Noneparasocial			−0.128
			(0.127)
Constant	1.417^**^	0.289	0.089
	(0.374)	(0.351)	(0.403)
Observations	230	230	230
*R*-squared	0.216	0.411	0.414
*F*-test	0	0	0
r2_a	0.184	0.384	0.384
*F*	6.751	15.27	13.98

*N = 230, Values in parentheses refers to standard errors.*

** *p < 0.01,*

* *p < 0.05. nonepara-Product of negative events and pseudo-social relations*.

H6 and H9 proposed a connection between the dimension of relational capital and purchase intention. The hypothesis testing results of H6 showed that there was no significant relationship between the streamer's commitment and live streaming commerce viewers' purchase intention (β = −0.085*, SE* = 0.065*, t* = −1.31). The hypothesis testing results of H6 showed a positive and significant connection between consumer reciprocal promotion and purchase intention in live streaming commerce (β = 0.146*, SE* = 0.059*, p* < 0.05). H7 proposed that the streamer's negative events are negatively related to the live streaming viewer's purchase intention. The hypothesis testing results are shown (β = −0.221, *SE* = 0.097, *p* < 0.05). H8 proposed that the streamer's parasocial relationship will negatively regulate the influence of the streamer's negative events on the purchase intention of live streaming viewers. Although Model 1 had significant regression results for negative events and Model 2 also had significant regression results on negative events and parasocial relationships, the interaction regression results between negative events and parasocial relations in Model 3 (β = −0.128*, SE* = 0.127, *t* = −1.01) were not significant, indicating that parasocial relations did not have a moderating effect on negative events and purchase intentions.

#### Mediating Effect Test

H3 and H5 proposed that trust has an intermediary effect in the influence of the streamer's parasocial relationship and professionalism on the viewer's purchase intention, respectively. This study adopted the Bootstrap method of indirect effects to directly test the mediation effect of ab (Preacher and Hayes, 2008). The Bootstrap method has no prerequisite requirements for the normality of the sampling distribution. The test principle is to randomly select the number of samples from the original samples to iterate and calculate an introductory effect with a new bootstrapped sample. At the same time, a 95% CI of the deviation correction is calculated. If the interval does not contain zero, it means that a mediating effect is significant. The results of testing an indirect influence of the streamer's parasocial relationship and professionalism on the viewer's purchase intention through a trust using the Bootstrap method for 5,000 iterations are shown in Table 4.

**Table 4:** Bootstrap analysis of trust mediation effect.

	**Observed'** **Coef.**	**Bootstrap** **SE**	**Normal-based** **[95% Conf. interval]**	
**Parasocial relationship and purchase intention**				
Indirect effect (ab)	0.170^**^	0.050	0.072	0.268
Direct effect (c')	0.420^**^	0.092	0.240	0.600
**Professionalism and Purchase Intention**				
Indirect effect (ab)	0.031^**^	0.011	0.010	0.053
Direct effect (c')	0.016	0.026	−0.034	0.067

*N = 230.*

** *p < 0.01*.

From the results of an analysis in Table 4, it can be seen that the indirect influence of trust on the streamer's parasocial relationship and the viewer's purchase intention was positive and significant. The 95% bias correction CI was (0.072, 0.368) excluding zero, and the mediation effect (ab) was 0.170. The direct effect (*c*') of the parasocial relationship on purchase intention was 0.420, and the 95% bias-modified CI is (0.240, 0.600) and excluding 0. After controlling the intermediate variable trust, a direct influence of the independent variable parasocial relationship on purchase intention was still significant. Therefore, trust played a partial mediating role between parasocial relationship and purchase intention. The mediation effect accounted for 40.48% of a direct effect and 28.81% of the total effect. An indirect influence of trust on the streamer's professionalism and the viewer's purchase intention was also positive and significant. The 95% deviation correction CI was (0.010, 0.053) excluding zero, and a mediating effect (ab) was 0.031. A direct effect (*c*') of professionalism on purchase intention was 0.016. The 95% deviation-modified CI was (−0.034, 0.067) including zero. That is, after controlling the intermediate variable trust, a direct effect of the independent variable professionalism on purchase intention was not significant. Therefore, trust played a completely intermediary role between professionalism and purchase intention. In summary, the Bootstrap test supported the mediation hypothesis proposed in this study.

## Discussion and Implications

The social capital theory explains the nature of personal emotions and the quality of relationships in social networks. As the linker of the virtual network, the purpose of this research was whether the streamer could find the main reason for the live purchase from social capital. The findings support the theoretical model of social capital and also strongly support the most hypothetical relationships. The findings showed that the streamer's centrality level, which means that the scale of live streaming e-commerce could not increase viewers' purchase intention. The results were inconsistent with our general understanding. In reality, the larger the scale of live streaming, the better the sales of live streaming. However, this seems to give us a revelation from another point of view. The larger the scale of live streaming, the larger the base of the viewer. Under the same purchase ratio, the number of viewer purchases will be higher. Because the data reflected the self-understanding of live broadcast viewers, it also reflected that viewers did not agree that the scale of live broadcast promoted their purchases. This fact was also confirmed by the feedback of consumers who often watch live broadcasts: people who watch live broadcasts will not buy becasue the number of people in the live streaming room is large, and more consideration was about viewers' own needs and the streamer's description of products.

Both the centrality of the streamer and the parasocial relationship of the viewer to the streamer can affect the scale of live streaming e-commerce, but the findings showed that the parasocial relationship of the viewer to the streamer significantly positively affects the viewer's purchase intention. Trust plays a part of the intermediary role between the two variables. This result confirmed that people with high viewer groups such as streamers, celebrities, and internet celebrities can achieve higher sales. When the streamer establishes personal popularity and gains the viewer's virtual intimacy recognition, live streaming viewers will trust the streamers that they believe to have a parasocial relationship, which will help promote product sales. Therefore, from the perspective of structural capital, the higher the streamer's centricity does not mean that it will increase the viewer's purchase intentions. However, if a trust relationship with viewers can be established outside live streaming, the streamer's centrality is similar to the result of parasocial relationship, which can effectively enhance the consumer's purchase intention. Therefore, if the streamer wants to improve the sales performance of their live streaming room, they can consider measures to establish an effective trust relationship with consumers outside the live streaming room.

The findings showed that the impact of negative events on viewers' purchase intentions was consistent with the real ones. If the streamer has a negative public event, it will significantly affect the viewer's purchase intentions. When the streamer's reputation is damaged, the viewer's purchase intentions are still greatly reduced due to the impact of such negative events, and they even arise not to watch the streamer's live show. Although it seems that the “star effect” makes viewers with parasocial relations adopt thinking methods such as problem transfer to weaken the influence of negative events on purchase intentions, the actual result has the opposite situation. That is, consumers' parasocial relations still cannot compensate for a negative impact of negative events, which means that even if the streamer is a public figure or celebrity, the audience still cannot tolerate their negative events.

The findings also showed that the professional performance of the streamers in cognitive capital significantly improved viewers' purchasing intentions. Trust played a full intermediary role in professionalism and purchasing intentions. Streamers' experience in sales practice is an important predictor of consumer purchases. Since live streaming commerce is still a sales task, it tests the streamer's sales ability. The more professional the streamer is, the more the streamer's introduction to the product will be recognized and trusted by the live viewer because viewers believe that streamers are experienced in their careers. Only when viewers trust the streamer's description of the product's functions and various ways of its use, as well as the streamer's reasons for recommending the product, will they be an intention to buy.

Contrary to expectations, the results showed that high-level relationship capital did not fully reflect the increase in viewers' purchase intention. There was a significant positive relationship between reciprocity expectations and the viewer's purchase intention. Promises are not related to purchase intentions. As the Chinese saying goes, “One spit, one nail,” “A word is once spoken cannot be overtaken even by a team of four horses,” which means that “if you promise somebody, you must keep it” or “a promise is weightier than one thousand bars of gold.” Therefore, the promise is generally a technique used by the streamers, and it can effectively enhance the trust of viewers and stimulate their enthusiasm for buying. It was a surprising result that the streamer's commitment during the live broadcast process does not affect the viewer's purchase intention. From another point of view, it also showed that the viewer was rational. The promise is regarded as the streamer's sales technique, and the streamer's promise is not recognized by the viewers, so it is not used as a reference for purchases. From the perspective of various promotions, discounts, and full reduction activities of current e-commerce, low prices are still one of the important factors for consumers to make online purchases, so reciprocity is called one of the main factors that effectively promote consumer purchases. This study did an additional analysis by removing the reciprocity variable from the model and checking whether the impact effect of the commitment exists. The results found that there was a weak positive correlation between the streamer's commitment and the viewer's purchase intentions, indicating that the irrelevant variance of reciprocity was suppressed, which slowed down the relationship between the commitment and the dependent variable. Once the impact of reciprocity is considered, higher commitments have little effect on purchase intention. A potential explanation for this discovery may be that, after taking into account the reciprocity factor, viewers are looking forward to preferential prices, but they believe that this is just a consistent routine between merchants and streamers. It was only determined in advance for live streaming sales, let alone the streamer's commitment. This pinpoints the direction of the streamer's behavior during live streaming: the joint promotion of low prices and promises is a more conducive way to promote sales.

## Limitations and Future Research

This study has several limitations, which need to be addressed. First, this research was a cross-sectional study based on the questionnaire survey, the independent and dependent variables in this study were all self-evaluated data from viewers. Due to a cross-sectional design, it was difficult to examine a dynamic interaction between the changes in the streamer's social capital and the resulting dynamic interaction effects in the viewer's purchase intentions. Therefore, future research can focus on the comparative relationship between longitudinal data.

Second, this study did not consider the relationship between the streamer's centrality and the parasocial relationship but regarded the scale of live streaming e-commerce brought about by the streamer's parasocial relationship as a manifestation of centrality. This expression is not very rigorous and becomes the limitation of this research. Future research can consider exploring the mutual influencing factors of streamer centrality and parasocial relationship and find the key path from centrality to establishing a trust relationship. However, the limitation of this research is that the streamer's professionalism was based on the self-assessment of viewers, which shows the consumer's understanding of the streamer's professionalism. The way of consumer evaluation lacks reasonable and comprehensive objective indicators to measure the streamer's professionalism, which needs to be objectively defined in further research.

Third, the streamer's parasocial relationship was determined by using theory as an independent variable in the model. However, parasocial relationship can also be regarded as a dependent variable or the result of the social capital model. Although parasocial relationship is an important indicator to promote viewers' purchase intention, the measurement of parasocial relationship may also be used to show the behavioral results achieved by the streamer's social capital. Therefore, future research should consider the dynamic nature of parasocial relationship and the social capital model. Of course, this study may be restricted due to sample selection and do not focus on fan groups with “star effects,” but this still does not prevent this conclusion from becoming a factor that should be considered. Future research can obtain sample data from the fan group for analysis to truly reflect the “star effect” on the mitigation of the streamer's negative events.

In addition, the limitation of this research was it only examined the factors that increase the viewer's purchase intention. Future research should examine whether the other sales models that rely on social network influence also exhibit similar factors, to see whether there are similar results of personal social capital found in this research. That is, whether the social capital model applies to the sales practices that are similar in nature.

## Conclusion

Although the development of e-commerce has brought people efficiency and convenience, huge and mixed information of online sales also makes people at a loss. A large amount of information about the products sold online also exacerbates the asymmetry of consumers' perception of product information. Live streaming commerce uses “people” as the link to eliminate a unilateral display of products sold in the past. The streamer's “lively” interactive display mechanism makes up for the information dissemination method of e-commerce unilaterally delivering product information. The connection between the social capital of the streamer's “individual” and the viewers' “crowd” creates a trustworthy image for consumers, effectively reducing the information asymmetry caused by consumer online purchases, thereby promoting consumers' purchase intentions.

Why do people trust the inaccessible and unfamiliar streamers through live streaming commerce to purchase online? The research findings show that when the streamer had a high professional ability in sales, low-price reciprocity norms during the live broadcast process and a parasocial relationship with the viewers, who watch the live broadcast show a strong willingness to buy. Surprisingly, the scale of the live broadcast by the streamer and the performance of its commitment to the viewer does not seem to promote consumers' purchase intentions. Live streaming commerce is a new form of e-commerce platform, and there is still a lot of room for the improvement of its consumer groups. How to fully show a unique, charm, and stable mechanism to attract people to use live streaming commerce as an online purchase option needs further academic exploration.

## Data Availability Statement

The raw data supporting the conclusions of this article will be made available by the authors, without undue reservation.

## Ethics Statement

Ethical review and approval was not required for the study on human participants in accordance with the local legislation and institutional requirements. Written informed consent for participation was not required for this study in accordance with the national legislation and the institutional requirements. Written informed consent was obtained from the individual(s) for the publication of any potentially identifiable images or data included in this article.

## Author Contributions

All authors listed have made a substantial, direct, and intellectual contribution to the work and approved it for publication.

## Funding

Research project “Research on the Economic Effects of Vocational Education Innovation Supply in the Guangdong-Hong Kong-Macao Greater Bay Area Based on the Rural Revitalization Strategy” (No. 2021ZDZX4070) from the Department of Education of Guangdong Province of China; 2021 Guangdong Province Educational Science Planning Special Project for Higher Education (No. 2021GXJK619); The start-up funding for Ph.D. scientific research of Hubei Normal University of China (No. 03106098); Research project “Relationship Between Customers Satisfaction and the Online Sales of Tourism Products in 7-11 Application” supported by Panyapiwat Institute of Management of Thailand in 2021; Social Science Planning Youth Project in Anhui Province of China (No. AHSKQ2021D34).

## Conflict of Interest

The authors declare that the research was conducted in the absence of any commercial or financial relationships that could be construed as a potential conflict of interest.

## Publisher's Note

All claims expressed in this article are solely those of the authors and do not necessarily represent those of their affiliated organizations, or those of the publisher, the editors and the reviewers. Any product that may be evaluated in this article, or claim that may be made by its manufacturer, is not guaranteed or endorsed by the publisher.
