# Supplementary material for: Influence of Streamer's Social Capital on Purchase Intention in Live Streaming E-Commerce
Source: Front Psychol. 2022 Jan 24;12:748172. doi: 10.3389/fpsyg.2021.748172 (PMC8819172; doi:10.3389/fpsyg.2021.748172)
Supplement: Supplementary file 1 [file Table_1.docx]

直播带货调研问卷

一、人口特征部分

1.您的性别是？

（1）男（2）女

2.您的年龄？

（1）20岁及以下（2）21-30岁（3）31-40岁（4）41-50岁 （5）50岁以上

3.您的教育水平是？（

（1）高中及以下 （2）专科 （3）本科 （4）硕士 （5）博士

4.您的职业类别是？

（1）专业技术人员（教师/医生/律师等） （2）服务业人员（餐饮服务员/司机/售货员等） （3）工人（工厂工人/建筑工人/城市环卫工人等） （4）公司职员 （5）事业单位/政府工作人员（6）学生 （7）自由职业者 （8）创业者

5.您的工作年限？

（1）0年 （2）0-3年 （3）3-5年 （4）5-8年 （5）8年以上

6.您的月可支配收入？

（1）1000元及以下 （2）1000-3000元 （3）3000-5000元 （4）5000-1万元 （5）1万以上

7.您一般是主要是在哪个直播平台上观看直播？

（1）微信直播（2）区域直播平台（非知名直播平台）（3）网易考拉直播（4）小红书直播（5）蘑菇街直播（6）抖音直播（7）快手直播 （8）淘宝直播（9）天猫直播（10）京东直播（11）拼多多直播（12）其他

二、结构资本

8.在您观看的直播带货中，一般有多少人次同时观看？

（1）100人以下 （2）100人至500人 （3）500-1000人 （4）1000人至3000人 （5）3000人至5000人 （6）5000人-1000人 （7）1万人以上

三、认知资本（专业性）

9.您认为主播对产品的讲解是否符合您对产品的认识？

（1）不符合 （2）不太符合 （3）一般 （4）比较符合 （5）非常符合

10.您认为主播对产品的讲解是为顾客着想的？

（1）不符合 （2）不太符合 （3）一般 （4）比较符合 （5）非常符合

四、关系资本（承诺）

11.主播在直播带货过程中出现“我保证”等词语的情况？

（1）没有出现 （2）偶尔出现 （3）一般 （4）较多出现 （5）经常出现

12.主播在直播过程中出现替产品商家做促销决定的情况？

（1）没有出现 （2）偶尔出现 （3）一般 （4）较多出现 （5）经常出现

13.主播认为他们带货商品是最低价的频率？

（1）没有出现 （2）偶尔出现 （3）一般 （4）较多出现 （5）经常出现

（互惠）

14.您在观看直播带货过程中主播推出抽奖等活动的频次？

（1）没有出现 （2）偶尔出现 （3）一般 （4）较多出现 （5）经常出现

15.您在观看直播带货过程中出现临时促销、低价的频次？

（1）没有出现 （2）偶尔出现 （3）一般 （4）较多出现 （5）经常出现

五、信任（认知信任）

16.主播的带货产品都是经过认真筛选的？

（1）不同意 （2）不太同意 （3）同意 （4）比较同意 （5）非常同意

17.主播在带货过程中不会出现弄虚作假行为？

（1）不同意 （2）不太同意 （3）同意 （4）比较同意 （5）非常同意

18.购买主播带货的产品我很放心，不用担心价格和售后等问题？

（1）不同意 （2）不太同意 （3）同意 （4）比较同意 （5）非常同意

19.主播对待直播带货工作是认真负责的？

（1）不同意 （2）不太同意 （3）同意 （4）比较同意 （5）非常同意

（情感信任）

220.我能够在直播过程中与主播自由的交流产品的看法和使用感受？

（1）不同意 （2）不太同意 （3）同意 （4）比较同意 （5）非常同意

21.主播在直播带货过程中投入了大量的感情能引起我的共鸣？

（1）不同意 （2）不太同意 （3）同意 （4）比较同意 （5）非常同意

22.我愿意在直播过程中与主播谈论带货产品的疑惑问题，并且知道主播能很好解决？

（1）不同意 （2）不太同意 （3）同意 （4）比较同意 （5）非常同意

23.我认为主播在带货过程中是将我们当作朋友来推荐产品的？

（1）不同意 （2）不太同意 （3）同意 （4）比较同意 （5）非常同意

六、拟社会关系

24.我觉得通过观看直播带货节目离主播的距离变得很近？（1）不同意 （2）不太同意 （3）同意 （4）比较同意 （5）非常同意

25.我非常喜欢观看主播的带货直播？

（1）不同意 （2）不太同意 （3）同意 （4）比较同意 （5）非常同意

26.我乐意观看主播在媒体上的各种信息？

（1）不同意 （2）不太同意 （3）同意 （4）比较同意 （5）非常同意

27.我觉得主播的直播带货过程很吸引人？

（1）不同意 （2）不太同意 （3）同意 （4）比较同意 （5）非常同意

28.当主播在直播过程中上犯错时，我比较心疼他（她）？

（1）不同意 （2）不太同意 （3）同意 （4）比较同意 （5）非常同意

29.我认为主播直播带货有助于提高我的购买的兴趣？

（1）不同意 （2）不太同意 （3）同意 （4）比较同意 （5）非常同意

七、负面事件

30.您经常观看的主播是否出现过直播带货的“翻车”事件，如产品与描述不符等负面行为？

（1）没有 （2）有

八、购买意向

31.直播过程中主播展示的产品能激发我的购买意向？

（1）不同意 （2）不太同意 （3）同意 （4）比较同意 （5）非常同意

32.我计划购买产品之前会先看主播产品直播后再做决定？

（1）不同意 （2）不太同意 （3）同意 （4）比较同意 （5）非常同意
