# Supplementary material for: Influence of Streamer's Social Capital on Purchase Intention in Live Streaming E-Commerce
Source: Front Psychol. 2022 Jan 24;12:748172. doi: 10.3389/fpsyg.2021.748172 (PMC8819172; doi:10.3389/fpsyg.2021.748172)
Supplement: Supplementary file 2 [file Table_2.docx]

Live Streaming E-commerce Questionnaire

1.Demographic part

1.1 Which is your gender?

（1）male （2）female

1.2 How old are you?

（1）Age 20 and under（2）21-30（3）31-40 （4）41-50 4（5）Over 50 years old

1.3 What is your education level?

（1）High school and below （2）college 2 （3）undergraduates （4）master （5）doctor

1.4 What is your occupational?

（1）Professional Position (teacher/doctor/lawyer, etc.)

（2）Service Position (catering waiter/driver/salesman, etc.)

（3）Worker (factory worker/construction worker/city sanitation worker, etc.)

（4）Corporate Employee

（5）Government Staff

（6）Student

（7）Freelancer

（8）Entrepreneur

1.5 How long do you have work experience?

（1）0 （2）0-3 years 2（3）3-5 years （4）5-8 years （5）over 8years

1.6 How much disposable income for you one month?

（1）1000 yuan and below （2）1000-3000 yuan （3）3000-5000 yuan （4）5000-10000 yuan （5）over 10000 yuan

1.7 Which live streaming platform do you usually watch?

（1）Wechat live streaming or Regional live streaming platform

（2）Live streaming of Koala，Xiaohongshu live or Mushroom Street live

（3）Jingdong live streaming, Pinduoduo live streaming or Microblog live streaming

（4）Tiktok live streaming or Kwai live streaming

（5）Taobao live streaming or Tmall live streaming

2. Structural capital（Centrality）

2.1 How many people do you watch the live streaming at the same time？

（1）Less than 100 people

（2）100 to 500

（3）500 to 1000

（4）1000 to 3000

（5）3000 to 5000

（6）5000 to 1000

（7）More than 10000

3. Cognitive capital（professional）

3.1 Do you think the streamer's explanation of the product is consistent with your understanding of the product? Do you think the anchor's explanation of the product is consistent with your understanding of the product?

（1）incompatible （2）Not very consistent（3）generally （4）More in line with （5）Very much in line

3.2 Do you think the streamer's explanation of the product is for the sake of customers?

（1）incompatible （2）Not very consistent（3）generally （4）More in line with （5）Very much in line

4. Relationship capital（commitment）

4.1 What happens to the words such as "I promise" when the streamer recommend the goods?

（1）Not present（2）Occasionally （3）generally （4）More appear（5）Often appear

4.2 Does the streamer make promotion decisions for merchants during the live streaming?

（1）Not present（2）Occasionally （3）generally （4）More appear（5）Often appear 4.3 The streamer thinks that the goods they recommend are the lowest frequency?

（1）Not present（2）Occasionally （3）generally （4）More appear（5）Often appear

5. Relationship capital（reciprocity）

5.1 How often do the streamer launch lottery and other activities when you watching the live streaming?

（1）Not present（2）Occasionally （3）generally （4）More appear（5）Often appear

5.2 How often do you have temporary promotions and low prices when watching live streaming?

（1）Not present（2）Occasionally （3）generally （4）More appear（5）Often appear

6.Trust（Cognitive trust）

6.1 The streamer's products are carefully screened?

（1）disagree （2）A little disagree（3）Agree（4）Quite agree（5）Strongly agree

6.2 Would the streamer not resort to fraud in the process of recommend goods?

（1）disagree （2）A little disagree（3）Agree（4）Quite agree（5）Strongly agree

6.3 I'm very relieved to buy the goods with streamer and don't worry about price and after-sales?

（1）disagree （2）A little disagree（3）Agree（4）Quite agree（5）Strongly agree

6.4 Is the streamer serious and responsible for the live streaming?

（1）disagree （2）A little disagree（3）Agree（4）Quite agree（5）Strongly agree

7.Trust（emotional trust）

7.1 Can I freely exchange my views and use feelings with the streamer during the live streaming?

（1）disagree （2）A little disagree（3）Agree（4）Quite agree（5）Strongly agree

7.2 The streamer has invested a lot of emotion in the process of live streaming, which can resonate with me?

（1）disagree （2）A little disagree（3）Agree（4）Quite agree（5）Strongly agree

7.3 I am willing to talk with the streamer about the doubts about the products with goods during the live streaming, and know that the anchor can solve them well?

（1）disagree （2）A little disagree（3）Agree（4）Quite agree（5）Strongly agree

7.4 I think the streamer takes us as friends to recommend goods in the process of live streaming?

（1）disagree （2）A little disagree（3）Agree（4）Quite agree（5）Strongly agree

8. Parasocial relationship

8.1 I think the distance from the streamier becomes very close by watching the live program with goods?

（1）disagree （2）A little disagree（3）Agree（4）Quite agree（5）Strongly agree

8.2 I like watching the streamer's live e-commerce very much?

（1）disagree （2）A little disagree（3）Agree（4）Quite agree（5）Strongly agree

8.3 I'd like to watch the streamer's various information on the media?

（1）disagree （2）A little disagree（3）Agree（4）Quite agree（5）Strongly agree

8.4 I think the live streaming process of the streamer is very attractive?

（1）disagree （2）A little disagree（3）Agree（4）Quite agree（5）Strongly agree

8.5 When the streamer makes mistakes in the live streaming, I am concerned about him (her)?

（1）disagree （2）A little disagree（3）Agree（4）Quite agree（5）Strongly agree

8.6 I think the streamer will help me increase my interest in buying?

（1）disagree （2）A little disagree（3）Agree（4）Quite agree（5）Strongly agree

9.Negative events

9.1 Have the streamer which you often watch ever experienced negative events with live streaming, such as negative behaviors such as inconsistent products and descriptions?

（1）Yes （2）No

10. Purchase intention

10.1 Could the products displayed by the streamer stimulate my purchase intention during the live streaming?

（1）disagree （2）A little disagree（3）Agree（4）Quite agree（5）Strongly agree

10.2 I plan to watch the live streaming of the streamer before making a decision?

（1）disagree （2）A little disagree（3）Agree（4）Quite agree（5）Strongly agree
